# CD8^+^ CD28^−^ regulatory T cells after induction therapy predict progression-free survival in myeloma patients: results from the GMMG-HD6 multicenter phase III study

**DOI:** 10.1038/s41375-024-02290-y

**Published:** 2024-06-03

**Authors:** Katharina Kriegsmann, Gigi Nu Hoang Quy Ton, Mohamed H. S. Awwad, Axel Benner, Uta Bertsch, Britta Besemer, Mathias Hänel, Roland Fenk, Markus Munder, Jan Dürig, Igor W. Blau, Stefanie Huhn, Dirk Hose, Anna Jauch, Christoph Mann, Niels Weinhold, Christof Scheid, Roland Schroers, Ivana von Metzler, Aneta Schieferdecker, Jörg Thomalla, Peter Reimer, Rolf Mahlberg, Ullrich Graeven, Stephan Kremers, Uwe M. Martens, Christian Kunz, Manfred Hensel, Andrea Seidel-Glätzer, Katja C. Weisel, Hans J. Salwender, Carsten Müller-Tidow, Marc S. Raab, Hartmut Goldschmidt, Elias K. Mai, Michael Hundemer

**Affiliations:** 1 Department of Internal Medicine V, Heidelberg University Hospital, Heidelberg, Germany; 2 Laborarztpraxis Rhein-Main MVZ GbR, Frankfurt am Main, Germany; 3 Division of Biostatistics, German Cancer Research Center (DKFZ) Heidelberg, Heidelberg, Germany; 4 National Center for Tumor Diseases Heidelberg, Heidelberg, Germany; 5 Department of Internal Medicine II, University Hospital Tübingen, Tübingen, Germany; 6 Department of Internal Medicine III, Klinikum Chemnitz, Chemnitz, Germany; 7 Department of Hematology, Oncology and Clinical Immunology, University Hospital Düsseldorf, Düsseldorf, Germany; 8 Department of Internal Medicine III, University Hospital Mainz, Mainz, Germany; 9 Department for Hematology and Stem Cell Transplantation, University Hospital Essen, Essen, Germany; 10 Medical Clinic, Charité University Medicine Berlin, Berlin, Germany; 11 Institute of Human Genetics, University of Heidelberg, Heidelberg, Germany; 12 Department of Hematology, Oncology and Immunology, Phillips-University Marburg, Marburg, Germany; 13 Department of Internal Medicine I, University Hospital Cologne, Cologne, Germany; 14 Department of Hematology, Oncology, Ruhr-University Bochum, Bochum, Germany; 15 Department of Internal Medicine II, University Hospital Frankfurt a.M., Frankfurt a.M., Germany; 16 Department of Oncology, Hematology and Bone Marrow Transplantation with Section of Pneumology, University Medical Center Hamburg-Eppendorf, Hamburg, Germany; 17 Hematology/Oncology Center, Koblenz, Germany; 18 Evang. Kliniken Essen-Mitte, Evang. Krankenhaus Essen-Werden, Essen, Germany; 19 Internal Medicine I, Hospital Mutterhaus der Borromäerinnen, Trier, Germany; 20 Department of Hematology, Oncology and Gastroenterology, Kliniken Maria Hilf GmbH, Mönchengladbach, Germany; 21 Hematology/Oncology Center, Lebach, Germany; 22 Hematology, Oncology, Palliative Care, SLK Clinics Heilbronn, Heilbronn, Germany; 23 Hematology and Oncology, Westpfalz-Klinikum, Kaiserslautern, Germany; 24 Mannheimer Onkologie Praxis, Mannheim, Germany; 25 Coordination Centre for Clinical Trials (KKS) Heidelberg, Heidelberg, Germany; 26 Asklepios Tumorzentrum Hamburg, Asklepios Hospital Hamburg Altona and St. Georg, Hamburg, Germany; 27 Molecular Medicine Partnership Unit (MMPU), University of Heidelberg and European Molecular Biology Laboratory (EMBL), Heidelberg, Germany

**Keywords:** Phase III trials, Myeloma

## To the editor:

In the battle against multiple myeloma (MM), T cell strategies have emerged as crucial, exploiting their natural tumor-cell targeting ability to enhance patient outcomes. Recent studies underscore the dual roles of T cells in MM: while specific T cells can effectively target and destroy MM cells, regulatory T cells may block this response, highlighting the complexity of the immune environment in MM [1, 2].

Elotuzumab, targeting the SLAM family member 7 (SLAMF7) protein, represents a promising advance enhancing natural killer (NK) cell activity against MM cells modulating T cell responses. This antibody not only boosts NK cell-mediated destruction of MM cells but also affects T cells, including a specific regulatory CD8^+^ subset, further contributing to its immunomodulatory effects [3–5]. Despite these promising mechanisms, clinical trials including the German-Speaking Myeloma Multicenter Group (GMMG)-HD6 study have yielded mixed results, highlighting the need for further investigation into its role in MM treatment [6–10].

This study aims to delve deeper into the impact of elotuzumab on T cell subsets within the MM microenvironment to elucidate its prognostic implications and refine therapeutic strategies. Within the context of the GMMG-HD6 trial, we performed a planned subgroup-analysis on SLAMF7 high expressing T cell subsets.

**Fig. 1:**
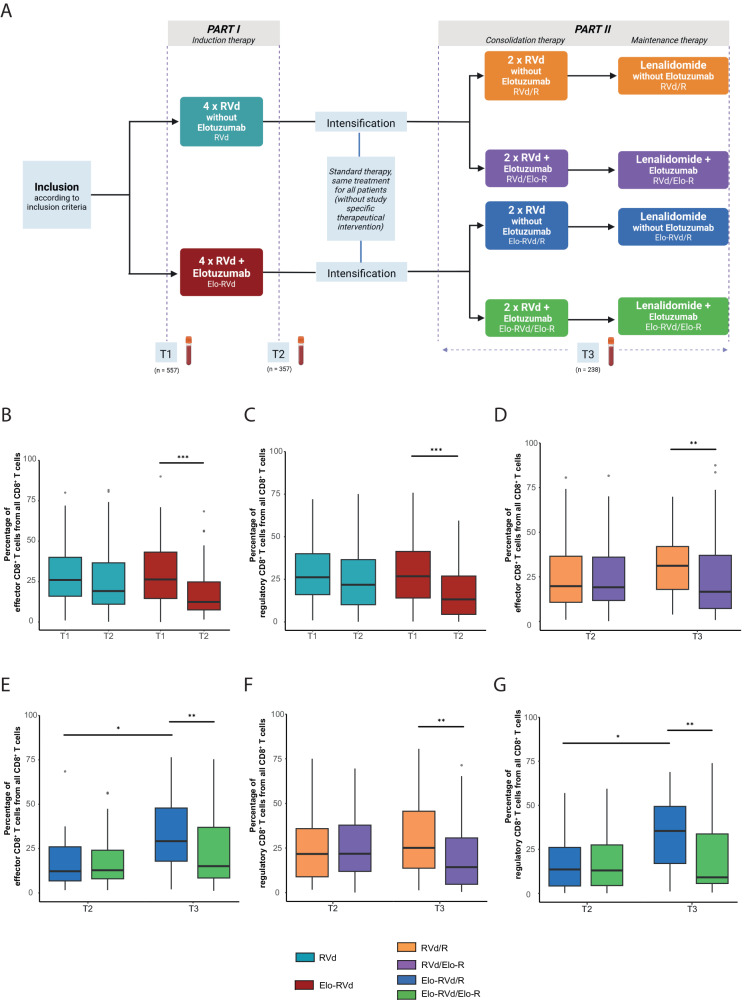
Box plots showing the percentages of the analyzed immune cell populations at inclusion (T1), after induction therapy (T2), and during consolidation and maintenance therapy (T3) by treatment arms. **A** Flow chart of the GMMG-HD6 trial and sample collection time points. MM patients were randomized into one of four arms (RVd/R, RVd/Elo-R, Elo-RVd/R, and Elo-RVd/Elo-R). Patients in the RVd/R or RVd/Elo-R arm received induction therapy consisting of four cycles of RVd. Patients in the Elo-RVd/R or Elo-RVd/Elo-R arm additionally received the monoclonal antibody elotuzumab in the four cycles of RVd. After induction therapy, patients underwent mobilization therapy followed by peripheral blood stem cell collection and melphalan high-dose chemotherapy/autologous stem cell transplantation. Consolidation therapy was performed with two cycles of RVd (RVd/R and Elo-RVd/R) or RVd/elotuzumab (RVd/Elo-R und Elo-RVd/Elo-R), followed by two years of lenalidomide maintenance therapy with elotuzumab for the RVd/Elo-R and Elo-RVd/Elo-R arms or without elotuzumab for the RVd/R and Elo-RVd/R arms. For the present study, peripheral blood samples were collected and analyzed at three different time points: at inclusion (T1), after induction therapy (T2), and during consolidation or maintenance therapy (T3). Box plots of the percentage of (**B**) effector CD8^+^ T cells and (**C**) regulatory CD8^+^ T cells (CD8^+^ CD28^-^) at T1 and T2 in study arm RVd/R + RVd/Elo-R and study arm Elo-RVd/R + Elo-RVd/Elo-R. **D**, **E** The percentage of effector CD8^+^ T cells at T2 and T3 in study arms RVd/R, RVd/Elo-R, Elo-RVd/R and Elo-RVd/Elo-R. **F**, **G** The percentage of regulatory CD8^+^ T (CD8^+^ CD28^-^) cells at T2 and T3 in study arms RVd/R, RVd/Elo-R, Elo-RVd/R and Elo-RVd/Elo-R. *Differences between groups were evaluated using Student’s t-test;* ∗ *p* < 0.05, ∗∗ *p* < 0.01, ∗∗∗ *p* < 0.001.

The GMMG-HD6 trial assessed elotuzumab combined with lenalidomide, bortezomib, dexamethasone (RVd) in newly diagnosed MM patients. Participants were allocated into four groups: RVd/R (RVd induction/consolidation plus lenalidomide maintenance), RVd/Elo-R (RVd induction, elotuzumab+RVd consolidation, and elotuzumab+lenalidomide maintenance), Elo-RVd/R (elotuzumab+RVd induction, RVd consolidation, lenalidomide maintenance), and Elo-RVd/Elo-R (elotuzumab+RVd for both induction/consolidation and elotuzumab+lenalidomide maintenance). Following induction, all underwent stem cell mobilization, high-dose melphalan, autologous stem cell transplantation, and two consolidation cycles, with 26 cycles of maintenance over three years (Fig. 1A). 564 patients were initially randomized in the trial. Five patients were excluded from the study due to violation of major eligibility criteria. The intention-to-treat (ITT) population of the study consisted of 559 patients. Peripheral blood (PB) samples for immune cell analysis were collected at baseline (T1) for 557 (99.6% ITT), post-induction (T2) for 357 (63.9% ITT), and during consolidation/maintenance (T3) for 238 (42.7% ITT) patients.

The baseline characteristics at T1 are depicted in Supplementary Table 1. The abundances of all analyzed immune cell types in each treatment arm are displayed in Supplementary Table 2 & Supplementary Fig. 1.

We recently demonstrated that elotuzumab induces Antibody Dependent Cellular Phagocytosis (ADCP) of SLAMF7 high regulatory CD8^+^ T cells in MM patients [5]. To confirm high SLAMF7 expression in specific subsets of CD8^+^ T cells within our study cohort, we performed flow cytometry analysis in representative PB/BM samples of newly diagnosed MM patients from different time points. The results confirmed the high SLAMF7 expression in the regulatory and effector CD8^+^ T cells in comparison to other CD8^+^ T cells (effector CD8^+^ T cells: *p* < 0.01 and regulatory CD8^+^ T cells *p* < 0.01; Supplementary Fig. 2A–D). Further, higher expression was observed across all time points, indicating the same distinctive phenotype (effector CD8^+^ T cells: *p* < 0.05 at all time points; regulatory CD8^+^ T cells: *p* < 0.05 at T1 and T3, *p* < 0.01 at T2; Supplementary Fig. 2E, F).

Our current analysis focused on differences induced by the variable expression of SLAMF7. We observed a decrease in effector CD8^+^ T cells with high SLAMF7 expression post induction treatment (T2) compared to baseline (T1) in treatment arms including Elo-RVd during induction therapy (26.3% [14.5–43.2%] vs. 12.4% [7.5–24.8%], *p* < 0.001; Fig. 1B, Supplementary Table 2 & Supplementary Fig. 3A). Similarly, we observed a decrease in regulatory CD8^+^ T cells with high SLAMF7 expression after induction treatment with Elo-RVd (26.8% [14.0–41.3%] vs. 13.2% [4.4%–26.9%], *p* < 0.001; Fig. 1C, Supplementary Table 2 & Supplementary Fig. 3B).

Based on a linear regression model, effector CD8^+^ T cells at T2 correlated with their initial level at T1 (*p* < 0.001) and elotuzumab-based induction treatment (*p* < 0.001) but not with age or sex (Supplementary Table 3). Regulatory CD8^+^ T cells at T2 correlated with their initial level at T1 (*p* < 0.001) and elotuzumab-based induction treatment (*p* < 0.001). In addition, female sex was negatively correlated with the levels of regulatory CD8^+^ T cells at T2 (*p* = 0.04; Supplementary Table 4).

Administration of elotuzumab during consolidation/maintenance (T3) without prior induction exposure (RVd/Elo-R) significantly reduced effector and regulatory CD8^+^ T cells with high SLAMF7 expression compared to patients not treated with elotuzumab (effector CD8^+^ T cells at T3: RVd/Elo-R 16.6% [7.3–37.0%] vs. RVd/R 31.2% [18.0–42%], *p* < 0.01 and regulatory CD8^+^ T cells at T3: RVd/Elo-R 14.3% [4.6–30.6%] vs. RVd/R 25.1% [13.7–45.6%], *p* < 0.01). Patients receiving elotuzumab during induction and consolidation/maintenance (i.e., Elo-RVd/Elo-R) maintained consistently lower levels of both cell types from T2 to T3 (effector CD8^+^ T cells at T2: 12.4% [7.5–24.8%] and at T3: 15.1% [8.4–36.9%], regulatory CD8^+^ T cells at T2: 13.2% [4.4–26.9%] and at T3: 9.1% [5.6–33.8%]; Fig. 1D–G, Supplementary Table 2 & Supplementary Fig. 3C–F).

Cessation of elotuzumab treatment during consolidation/maintenance after initial therapy during induction (i.e., Elo-RVd/R) resulted in a rise in the levels of effector and regulatory CD8^+^ T cells with high SLAMF7 expression compared to their T2 levels (effector CD8^+^ T cells at T2: 12.4% [7.5–24.8%] and at T3: 29.1% [17.9–47.8%], *p* < 0.05; regulatory CD8^+^ T cells at T2: 13.2% [4.4–26.9%] and at T3: 35.4% [16.9–49.4%], *p* < 0.05; Fig. 1D–G & Supplementary Table 2 & Supplementary Fig. 3C–F). Interestingly, we observed that the decrease in effector CD8^+^ T cells, caused by elotuzumab treatment, led to an increase in central memory CD8^+^ T cells at T2 and T3 (Supplementary Fig. 3G–J).

The optimal cut-off regarding progression free survival (PFS) for subdividing patients with regulatory CD8^+^ T cells with high versus low SLAMF7 expression levels at T2 was 20%. The application of a multivariable Cox proportional hazards model at the end of induction therapy (T2) identified a statistically significant prognostic effect determined by effector CD8^+^ T cells (HR = 0.88, 95% CI = 0.77-0.99), female sex (HR = 0.63, 95% CI = 0.42-0.94), and high regulatory CD8^+^ T cell levels for patients who received elotuzumab during consolidation and maintenance therapy (RVd/Elo-R: HR = 4.12, 95% CI = 1.78-9.63 and Elo-RVd/Elo-R: HR = 3.12, 95% CI = 1.39-7.01). Moreover, an interaction effect between treatment and regulatory CD8^+^ T cells at T2 was observed. A worse PFS time was observed for patients with high levels of regulatory CD8^+^ T cells when treated with elotuzumab-based consolidation/maintenance therapy compared to the standard treatment arm (high regulatory CD8^+^ T cells at T2: RVd/Elo-R vs. RVd/R HR = 2.19, 95% CI: 1.11-4.33 and Elo-RVd/Elo-R vs. RVd/R HR = 2.03, 95% CI: 0.98-4.20; Table 1). Corresponding landmark analyses of PFS for each treatment arm and regulatory CD8^+^ T cells at T2 are shown in Supplementary Fig. 4.

**Table 1:** Multivariable model on progression-free survival from landmark post induction therapy (T2).

Variable	Effect	Hazard ratio	95% Confidence limits	
**Age**	10 years increment	1.23	0.93	1.62
**Sex**	Female : Male	0.63	0.42	0.94
**R-ISS**	II : I	1.49	0.89	2.50
	III : I	1.46	0.68	3.12
	Not classified : I	0.95	0.37	2.44
**Effector CD8**^**+**^ **T cells at T1**	30% increase	0.88	0.77	0.99
**Regulatory CD8**^**+**^ **T cells at T2** High : low	RVd/R	1.02	0.45	2.29
	RVd/Elo-R	4.14	1.78	9.63
	Elo-RVd/R	1.11	0.51	2.43
	Elo-RVd/Elo-R	3.12	1.39	7.01
**Treatment arm** RVd/Elo-R : RVd/R	Low reg. CD8^+^ T cells	0.54	0.22	1.34
	High reg. CD8^+^ T cells	2.19	1.11	4.33
**Treatment arm** Elo-RVd/R : RVd/R	Low reg. CD8^+^ T cells	1.16	0.55	2.46
	High reg. CD8^+^ T cells	1.26	0.58	2.77
**Treatment arm** Elo-RVd/Elo-R : RVd/R	Low reg. CD8^+^ T cells	0.66	0.29	1.53
	High reg. CD8^+^ T cells	2.03	0.98	4.20

Multivariable Cox proportional hazards model on progression-free survival from landmark post induction therapy (T2) including 303 available patients with complete measurements of regulatory CD8^+^ T cells. Missing values were imputed for high-risk cytogenetics (included in R-ISS) and effector CD8^+^ T cells at T1. Cut-off (high vs. low) for regulatory CD8^+^ T cells at T2 was identified at 20% by maximally selected log-rank statistics. Hazard ratios and confidence intervals are computed for a 10% change in effector CD8^+^ cells, regulatory CD8^+^ T cells at T2 dependent on treatment arm, and separated by treatment arms based on low and high levels of regulatory CD8^+^ T cells at T2.

Despite the trial’s overarching findings, which did not demonstrate a PFS benefit from elotuzumab addition, our analysis pivoted towards dissecting the impact of SLAMF7-positive T-cell subsets on patient prognosis under elotuzumab therapy. We found that elotuzumab selectively targets and diminishes effector and regulatory CD8^+^ T cells marked by high SLAMF7 expression. Intriguingly, cessation of elotuzumab led to the resurgence of these cells, hinting at a complex interplay between treatment and immune recovery [5]. Moreover, increased effector CD8^+^ T cells with high SLAMF7 expression at baseline correlated with improved PFS, consistent with other studies [11, 12].

Our pivotal finding—that elevated levels of regulatory CD8^+^ T cells post-induction correlate with inferior PFS in patients receiving elotuzumab during consolidation/maintenance phases —illuminates a critical dimension of elotuzumab’s action mechanism. Our findings suggest two key insights into CD8^+^ T cell roles in MM treatment outcomes. First, the impact of regulatory CD8^+^ T cells with high SLAMF7 expression on PFS becomes significant only after induction therapy, indicating a dynamic interplay with other immune populations such as effector T cells. Second, these regulatory CD8^+^ T cells might identify patients who benefit more from specific immunomodulatory treatments like elotuzumab, especially during later treatment phases. This highlights the complexity of the immune response in treatment efficacy and suggests a path towards personalized therapy.

Recently, Li et al., which identified a subset of suppressive CD8^+^ T cells expressing killer cell immunoglobulin-like receptor (KIR) in the context of SARS-CoV-2, we explored the possibility of overlap with our identified regulatory CD8^+^ T cell population [13]. However, our analysis indicated only slight expression of KIR on the regulatory CD8^+^ T cells, suggesting that they are distinct (data not shown). Noteworthy, a similar CD8^+^ T cell populations have been characterized by Pangrazzi et al. to have senescent-like attributes [14]. Senescence in CD8^+^ T cells is typically associated with diminished proliferative potential and altered functional properties. Meanwhile, our previous findings demonstrated that these regulatory CD8^+^ T cells maintain potent suppressive functions within the context of MM, indicating an active, rather than senescent, state [15].

All in all, this study marks a significant step in understanding the immunological underpinnings of elotuzumab’s efficacy in MM therapy. The delineation of T cell subsets by SLAMF7 expression levels post-induction offers a novel prognostic tool, suggesting a more critical approach to leveraging immune dynamics in MM treatment strategies. However, such insights, derived from a multivariable analysis, require further exploration into how these immune landscapes might be navigated to enhance patient outcomes in the evolving landscape of MM therapy.

### Supplementary information

Supplemental material
