# Supplementary material for: CD8+ CD28− regulatory T cells after induction therapy predict progression-free survival in myeloma patients: results from the GMMG-HD6 multicenter phase III study
Source: Leukemia. 2024 Jun 3;38(7):1621–5. doi: 10.1038/s41375-024-02290-y (PMC11216978; doi:10.1038/s41375-024-02290-y)
Supplement: Supplementary file 1 — Supplemental material [file 41375_2024_2290_MOESM1_ESM.pdf]

$CD8^+$ $CD28^-$ Regulatory T cells after induction therapy predict progression-free survival in myeloma patients: Results from the GMMG-HD6 multicenter phase III study

Patients and methods 2
Supplementary Table 1: Baseline characteristics according to patients' sample availability at the analyzed time points. 6
Supplementary Table 2: Analyzed immune cell populations at different time points. 7
Supplementary Table 3: Linear regression model on factors that influence effector $CD8^+$ T cell levels at T2 11
Supplementary Table 4: Linear regression model on factors that influence regulatory $CD8^+$ T cell levels at T2 11
Supplementary Figure 1: Coordinate plots showing the percentages of T cell subpopulations and NK cells of each patient from inclusion (T1) to after induction therapy (T2) and from after induction therapy to consolidation and maintenance therapy (T3): 12
Supplementary Figure 2: Flow cytometry analysis of SLAMF7 expression in effector $CD8^+$ and regulatory $CD8^+$ T cells of MM patients: 17
Supplementary Figure 3: Dot plots and pie charts showing the percentages of effector $CD8^+$ T cells, regulatory $CD8^+$ T cells and other $CD8^+$ T cell subsets of MM patients from inclusion (T1), after induction therapy (T2) and during consolidation and maintenance therapy (T3) by treatment arms: 19
Supplementary Figure 4: Kaplan–Meier curves showing progression-free survival of patients, evaluating the abundance of regulatory $CD8^+$ ($CD8^+$ $CD28^-$) T cells after induction therapy (T2), improving the prediction of response to elotuzumab-based maintenance therapy: 21
References 26

Patients and methods

Patient cohort, sample collection and clinical variables

The GMMG-HD6 trial was an open-label, prospective, multicenter, randomized, parallel group, phase 3 clinical trial of the effect of elotuzumab in RVd induction/consolidation and lenalidomide maintenance on patients with newly diagnosed MM with the primary objective of determining the best of four treatment strategies regarding progression free survival (PFS) (NCT02495922) [1]. The primary results of the GMMG-HD6 trial were previously presented [2].

All patients gave written informed consent before participating in the study. The trial was conducted in accordance with the Good Clinical Practice Guidelines of the International Conference on Harmonization (ICH-GCP) and the ethical principles described in the Declaration of Helsinki. Approval was obtained from the ethics committee of the University of Heidelberg in collaboration with the participating centers' ethics committees. The trial was sponsored by the Heidelberg University Hospital (Heidelberg, Germany) and conducted by the GMMG (Heidelberg, Germany) and associated trial sites.

The present analysis represents a prespecified immunological study accompanying the GMMG-HD6 trial evaluating the prognostic effect of immune cell populations [1]. For this, peripheral blood (PB) samples from MM patients treated in the trial were collected and analyzed at inclusion (T1), after induction therapy (T2), and during consolidation or maintenance therapy (T3; samples were collected within 9 to 36 months post randomization; Figure 1A).

Trial treatment

The study randomized patients into four study arms: in the RVd/R arm, patients received lenalidomide, bortezomib, dexamethasone induction and consolidation as well as lenalidomide maintenance. In the RVd/Elo-R arm, patients received RVd induction, elotuzumab+RVd consolidation and elotuzumab+lenalidomide maintenance. Patients in the Elo-RVd/R arm received elotuzumab+RVd induction followed by RVd consolidation and lenalidomide maintenance. Patients in the Elo-RVd/Elo-R arm received elotuzumab+RVd induction and consolidation and elotuzumab+lenalidomide maintenance. After four cycles of induction therapy, patients received stem cell mobilization and collection, high-dose melphalan, and autologous hematopoietic stem cell transplantation according to GMMG standard protocols, followed by two cycles of consolidation treatment. Maintenance therapy was given for 26 cycles in three years.

Characterization of immune cell populations by flow cytometry

Fluorochrome-conjugated antibodies were used for NK and T-cell flow cytometry analysis. Anti-CD56-FITC (NCAM16), anti-CD3-PE-Cyanine7 (SK7), anti-CD45-APC-H7 (2D1), anti-CD45RA-FITC (HI100), anti-CD4-PerCP-Cyanine5 (SK3), and anti-CD28-PE (CD28.2) were purchased from BD Biosciences/Pharmingen, Heidelberg, Germany. Anti-CCR7-PacB (G043H7) and anti-CD57-FITC (HCD57) were purchased from BioLegend, Fell, Germany. Anti-CD3-PE (UCHT1), anti-CD8-PE-Cyanine7 (SFCI21Thy2D3), and anti-CD25-PE-Cyanine7 (A52882) were purchased from Beckman Coulter, Krefeld, Germany. Dimethylsulfoxide was purchased from Serva, Heidelberg, Germany, and Ficoll (Histopaque) from Sigma–Aldrich, Taufkirchen, Germany. Fetal bovine serum (FBS), RPMI 1640 medium with 2 mM L-glutamine and 10x phosphate-buffered saline (PBS) were purchased from Thermo Fisher, Darmstadt, Germany. Cell strainers (100 µm) from Greiner Bio-One, Frickenhausen, Germany, were used. Peripheral blood mononuclear cells were generated from PB samples by purification via a Ficoll gradient and frozen in FBS containing 10% DMSO. Samples were thawed, and thawing medium (10% FBS in RPMI 1640 cell medium) was added. Samples were centrifuged, resuspended in 1x PBS and filtered through a 100 µm cell strainer. Staining of cells with fluorochrome-labeled antibodies, including fluorescence-minus-one controls, was performed at room temperature for 15 min in the dark. Cells were washed 3 times with 1x PBS. Flow cytometry data were acquired on a FACS Canto cytometer (BD Biosciences, Heidelberg, Germany). The cell populations were defined and gated with BD FACSDiva v8.0.1 software (BD Biosciences, Heidelberg, Germany) as described in Supplementary Figure 5. Effector $CD8^+$ T cells were identified as $CCR7^-$ and $CD45RA^+$ cells and are reported as the percentages of all $CD8^+$ T cells. Regulatory $CD8^+$ T cells were identified as $CD28^-$ and $CD57^+$ cells and are reported as the percentages of all $CD8^+$ T cells.

For measuring SLAMF7 expression on effector and regulatory $CD8^+$ T cells in BM samples of newly diagnosed MM patients, as well as in PB patients samples at time points T1, T2, and T3, following fluorochrome-conjugated antibodies were used: Anti-CD45-APC/Cy7 (2D1), PerCP-CD8 (SK1), Alexa700-CD3 (UCHT1), BV421-CCR7 (G043H7) were purchased from BioLegend, Fell, Germany. Anti-SLAMF7-AlexaFluor647 (235614), anti CD45RA-FITC (HIT100), anti-CD28-PE (CD28.2) and anti-CD57-FITC (NK-1) were purchased from BD Biosciences, Heidelberg, Germany. Cells were stained as previously described and flow cytometry data were acquired on a FACS Lyrics cytometer (BD Biosciences, Heidelberg, Germany). Cell populations were defined as previously described using FlowJo™ v10 software (BD Biosciences, Heidelberg, Germany) and SLAMF7 expression was measured using median flow intensity (MFI) in effector and regulatory $CD8^+$

T cells and compared to the non-effector and non-regulatory $CD8^+$ T cell population respectively.

Molecular cytogenetic testing

Molecular cytogenetic testing was performed using a previously described method [3]. Briefly, $CD138^+$ BM plasma cells were purified using automagnetic-activated cell sorting with anti-CD138 immunobeads. For interphase fluorescence in situ hybridization analyses, a panel of two-color probe sets was used to detect numerical changes at the chromosomal loci 1q21/13q14, 5p15/5q35, 8p21/19q13, 9q34/15q22, and 11q22.3/17p13 as well as the immunoglobulin heavy locus (IgH)-translocations t(11;14)(q13;q32), t(4;14)(p16;q32), t(14;16)(q32;q23), or any other IgH-rearrangement. Hybridization was performed according to the manufacturer's instructions (MetaSystems, Altlussheim, Germany and Cytocell, Cambridge, UK), and a minimum of 100 interphase nuclei per probe were evaluated using an automated spot counting system (Applied Spectral Imaging, Edingen-Neckarhausen, Germany). Hybridization efficiency was validated using interphase nuclei obtained from the BM of a healthy donor, and the thresholds for gains, deletions, and translocations were set at 10%. Hyperdiploidy was defined according to the criteria described by Wuilleme et al., which require trisomy of at least two of the three chromosomes 5, 9, and 15 [4]. High-risk cytogenetics was defined by the presence of deletion 17p and/or t(4;14) or t(14;16) [5].

Statistical analysis

The main objective of this analysis was the characterization of patients´ immune cell populations throughout the trial. Box plots of individual cell populations indicate median values, interquartile ranges (q1 – q3), and outliers. Multivariable linear regression models were applied to investigate the influence of induction treatment on T-cell populations at T2. The covariates used were T-cell subpopulation data at T1 (per 10%), age (per 10 years), sex (female vs. male), and treatment arm (Elo-RVd vs. RVd induction therapy).
A two-stage procedure was used, consisting of a cutoff test by maximally selected log-rank statistics [6]followed by multivariable Cox proportional hazards models using the post induction (T2) landmark. Multivariable Cox proportional models were applied to assess PFS to investigate the influence of induction treatment on T cell populations at T2 (end of induction). Covariates comprised age (per 10 years), sex (female vs. male), revised International Staging System (R-ISS; stage II vs. I and III vs. I), effector $CD8^+$ T cells (at T1; per 10% increase), regulatory $CD8^+$ T cells (at T2; high vs. low), and treatment arm (Elo-RVd/R vs. RVd/R, RVd/Elo-R vs. RVd/R, and Elo-RVd/Elo-R vs. RVd/R). The results are illustrated by hazard ratios (HRs) together with 95% confidence intervals (95% CIs).

The median duration of follow-up was calculated using the reverse Kaplan–Meier estimate, and the Kaplan–Meier method was used to estimate time-to-event distributions [7]. For all hypotheses, a p value smaller than 5% denotes a statistically significant result. All statistical analyses were performed with the statistical software environment R, version 4.0.4 (Vienna, Austria), using the R packages survival, version 3.2-7, and prodlim, version 2019.11.13.

Supplementary Table 1: Baseline characteristics according to patients' sample availability at the analyzed time points.

Variable (at baseline)		At inclusion (T1) (n = 557)	After induction therapy (T2) (n = 357)	During consolidation or maintenance therapy (T3) (n = 238)
		n (%)	n (%)	n (%)
Treatment Arm	RVd/R	139 (25.0)	88 (24.7)	50 (21.0)
	RVd/Elo-R	140 (25.1)	94 (26.3)	64 (26.9)
	Elo-RVd/R	137 (24.6)	87 (24.4)	60 (25.2)
	Elo-RVd/Elo-R	141 (25.3)	88 (24.6)	64 (26.9)
Age (years)	median (range)	59 (27 – 70)	59 (27 – 70)	59 (27 – 70)
Sex	Male	315 (56.5)	204 (57.1)	133 (55.9)
	Female	242 (43.5)	153 (42.9)	105 (44.1)
WHO performance status	0-1	506 (90.8)	328 (91.9)	222 (93.3)
	>1	50 (9)	29 (8.1)	16 (6.7)
	Missing	1 (0.2)	-	-
LDH	Above upper limit of normal	92 (16.5)	57 (16.0)	37 (15.5)
	Below upper limit of normal	462 (83.0)	300 (84.0)	201 (84.5)
	Missing	3 (0.5)	-	-
High risk cytogenetics	Yes	119 (21.4)	82 (23.0)	45 (18.9)
	No	338 (60.7)	215 (60.2)	149 (62.6)
	Missing	100 (17.9)	60 (16.8)	44 (18.5)
ISS	I	227 (40.8)	153 (42.9)	95 (39.9)
	II	205 (36.8)	129 (36.1)	94 (39.5)
	III	125 (22.4)	75 (21.0)	49 (20.6)
R-ISS	I	133 (23.9)	90 (25.2)	56 (23.5)
	II	319 (57.3)	208 (58.1)	141 (59.2)
	III	56 (10.0)	32 (9.0)	19 (8.1)
	Not classified	49 (8.8)	27 (7.6)	22 (9.2)

ISS and R-ISS were defined according to [8,9].
High risk cytogenetic was defined as del 17p and/or t(4;14) and/or gain of 1q21 (>3 copies).

(R-) ISS, (Revised) International Staging System; LDH, lactate dehydrogenase; WHO, World Health Organization.

Supplementary Table 2: Analyzed immune cell populations at different time points.

Immune cell population	Immune marker profile	Induction treatment arm	At inclusion (T1)			After induction therapy (T2)			Treatment arm	During consolidation or maintenance therapy (T3)		
			n	NA	Median (q1; q3)	n	NA	Median (q1; q3)		n	NA	Median (q1; q3)
**A. Lymphocytes, T cells and NK cells**												
**Lymphocytes (% of nucleated cells)**	side scatter low, $CD45^+$, excluding debris/doublets	RVd	172	107	50.9 (35.8; 73.2)	165	114	36.4 (18.0; 57.2)	RVd/R	37	102	25.8 (21.9; 44.0)
									RVd/Elo-R	56	84	20.0 (7.5; 33.0)
		Elo-RVd	177	101	56.0 (31.0; 77.0)	166	112	31.9 (16.9; 58.3)	Elo-RVd/R	56	81	30.1 (17.1; 41.5)
									Elo-RVd/Elo-R	57	84	27.4 (13.3; 41.4)
		All	349	208	52.1 (33.1; 74.0)	331	226	34.4 (17.2; 58.2)	All	206	351	25.4 (14.3; 40.6)
**T cells (% of lymphocytes)**	$CD3^+$ of lymphocytes	RVd	172	107	78 (70.0; 84.0)	165	114	80.9 (73.7; 87.7)	RVd/R	37	102	59.4 (50.0; 67.5)
									RVd/Elo-R	56	84	47.8 (25.3; 56.4)
		Elo-RVd	177	101	79.0 (70.0; 85.0)	166	112	85.4 (79.1; 90.0)	Elo-RVd/R	56	81	53.5 (38.6; 67.9)
									Elo-RVd/Elo-R	57	84	55.2 (39.9; 65.2)
		All	349	208	78.0 (70.0; 84.0)	331	226	83.2 (75.8; 88.9)	All	206	351	52.2 (35.7; 65.0)
**NK cells (% of lymphocytes)**	$CD3^-$, $CD56^+$ of lymphocytes	RVd	172	107	10.6 (7.3; 16.2)	165	114	13.0 (9.2; 19.4)	RVd/R	36	103	32.8 (25.1; 49.1)
									RVd/Elo-R	56	84	44.8 (28.7; 59.4)
		Elo-RVd	173	105	10.7 (7.2; 17.4)	164	114	9.7 (6.2; 14.7)	Elo-RVd/R	56	81	35.9 (20.9; 47.0)
									Elo-RVd/Elo-R	56	85	37.3 (23.6; 49.7)
		All	345	212	10.7 (7.3; 16.7)	329	228	11.0 (7.3; 17.2)	All	204	353	37.3 (23.4; 51.4)
**B. $CD4^+$ T cells and their subpopulations**												
**$CD4^+$ T cells (% of T cells)**	$CD4^+$ of T cells	RVd	172	107	78.0 (70.0; 84.0)	165	114	80.9 (73.7; 87.7)	RVd/R	37	102	42.1 (29.1; 49.0)
									RVd/Elo-R	56	84	49.8 (33.6; 63.4)
		Elo-RVd	177	101	79.0 (70.0; 85.0)	166	112	85.4 (79.1; 90.0)	Elo-RVd/R	55	82	41.4 (27.2; 51.0)
									Elo-RVd/Elo-R	57	84	48.8 (32.8; 59.6)

		All	349	208	78.0 (70.0; 84.0)	331	226	83.2 (75.8; 88.9)	All	205	352	45.7 (31.0; 55.7)
**Central memory CD4⁺ T cells (% of CD4⁺ T cells)**	$CCR7^+$, $CD45RA^-$ of $CD4^+$ T cells	RVd	166	113	36.0 (28.0; 44.0)	163	116	35.5 (29.0; 44.9)	RVd/R	37	102	30.9 (23.8; 37.6)
									RVd/Elo-R	56	84	29.2 (24.2; 35.6)
		Elo-RVd	177	101	35.0 (29.0; 44.0)	167	111	34.3 (27.2; 43.2)	Elo-RVd/R	56	81	28.3 (23.7; 36.2)
									Elo-RVd/Elo-R	57	84	30.5 (25.5; 36.9)
		All	343	214	36.0 (28.4; 44.0)	330	227	35.2 (28.2; 44.1)	All	206	351	29.9 (24.3; 36.6)
**Effector memory CD4⁺ T cells (% of CD4⁺ T cells)**	$CCR7^-$, $CD45RA^-$ of $CD4^+$ T cells	RVd	166	113	7.6 (4.0; 12.0)	163	116	14.1 (10.2; 20.3)	RVd/R	37	102	48.0 (38.1; 53.2)
									RVd/Elo-R	56	84	46.0 (38.2; 54.8)
		Elo-RVd	177	101	9.0 (4.0; 15.8)	167	111	15.2 (9.9; 21.2)	Elo-RVd/R	56	81	42.1 (34.4; 53.0)
									Elo-RVd/Elo-R	57	84	43.3 (32.9; 51.0)
		All	343	214	8.0 (4.0; 14.0)	330	227	14.8 (10.0; 20.6)	All	206	351	44.0 (35.3; 53.4)
**Naïve CD4⁺ T cells (% of CD4⁺ T cells)**	$CCR7^+$, $CD45RA^+$ of $CD4^+$ T cells	RVd	166	113	48.0 (37.0; 61.9)	163	116	43.2 (32.3; 53.9)	RVd/R	37	102	11.7 (6.6; 17.8)
									RVd/Elo-R	56	84	12.6 (8.8; 20.0)
		Elo-RVd	177	101	45.6 (36.0; 60.0)	167	111	44.4 (33.4; 55.4)	Elo-RVd/R	56	81	13.1 (7.4; 24.7)
									Elo-RVd/Elo-R	57	84	14.3 (10.2; 23.9)
		All	343	214	46.0 (36.2; 61.0)	330	227	43.9 (32.7; 54.8)	All	206	351	13.1 (7.8; 21.2)
**Effector CD4⁺ T cells (% of CD4⁺ T cells)**	$CCR7^-$, $CD45RA^+$ of $CD4^+$ T cells	RVd	166	113	2.0 (1.0; 5.0)	163	116	2.0 (1.1; 4.9)	RVd/R	37	102	3.9 (3.1; 11.7)
									RVd/Elo-R	56	84	4.9 (2.6; 9.3)
		Elo-RVd	177	101	2.0 (1.0; 5.0)	167	111	1.9 (0.8; 4.2)	Elo-RVd/R	56	81	5.8 (3.5; 16.5)
									Elo-RVd/Elo-R	57	84	4.5 (2.2; 10.7)
		All	343	214	2.0 (1.0; 5.0)	330	227	1.9 (0.9; 4.5)	All	206	351	4.9 (2.7; 11.1)
**Regulatory CD4⁺ T cells (% of CD4⁺ T cells)**	$CD25^+$ of $CD4^+$ T cells	RVd	165	114	3.0 (1.9; 4.6)	164	115	5.6 (3.4; 9.4)	RVd/R	37	103	32.2 (24.5; 51.2)
									RVd/Elo-R	56	84	42.1 (24.7; 63.4))
		Elo-RVd	172	106	3.0 (2.0; 5.2)	165	113	5.0 (2.9; 8.5)	Elo-RVd/R	55	81	29.3 (19.3; 45.4)
									Elo-RVd/Elo-R	57	85	41.5 (26.9; 58.9)

		All	337	220	3.0 (1.9; 5.0)	329	228	5.3 (2.9; 9.2)	All	205	353	37.2 (23.0; 57.7)
**C. CD8⁺ T cells and their subpopulations**												
**CD8+ T cells (% of T cells)**	CD8⁺ of T cells	RVd	171	108	28.0 (20.8; 36.0)	165	114	30.7 (22.7; 41.7)	RVd/R	37	102	57.9 (51.0; 70.9)
									RVd/Elo-R	56	84	50.2 (36.6; 66.4)
		Elo-RVd	177	101	31.0 (21.0; 39.0)	167	111	28.6 (20.1; 37.7)	Elo-RVd/R	56	81	56.8 (48.5; 72.7)
									Elo-RVd/Elo-R	57	84	51.2 (40.4; 67.2)
		All	348	209	29.4 (21.0; 38.0)	332	225	29.4 (21.2; 40.6)	All	206	351	54.0 (44.4; 68.9)
**Central memory CD8⁺ T cells (% of CD8⁺ T cells)**	CCR7⁺, CD45RA⁻ of CD8⁺ T cells	RVd	165	114	13.0 (8.0; 20.0)	163	116	18.8 (11.5; 27.1)	RVd/R	37	102	12.7 (8.8; 22.5)
									RVd/Elo-R	56	84	20.1 (10.3; 31.8)
		Elo-RVd	177	101	13.0 (8.0; 19.0)	167	111	22.5 (15.0; 29.5)	Elo-RVd/R	56	81	10.8 (6.5; 16.2)
									Elo-RVd/Elo-R	57	84	20.6 (12.7; 30.5)
		All	342	215	13.0 (8.0; 19.0)	330	227	20.5 (12.9; 28.3)	All	206	351	15.5 (9.1; 27.1)
**Effector memory CD8⁺ T cells (% of CD8⁺ T cells)**	CCR7⁻, CD45RA⁻ of CD8⁺ T cells	RVd	165	114	20.0 (13.0; 30.0)	163	116	27.6 (17.9; 40.5)	RVd/R	37	102	41.2 (33.9; 56.3)
									RVd/Elo-R	56	84	37.0 (21.0; 53.1)
		Elo-RVd	176	102	19.0 (13.0; 30.0)	167	111	31.1 (20.7; 40.9)	Elo-RVd/R	56	81	40.6 (27.1; 53.0)
									Elo-RVd/Elo-R	57	84	40.2 (25.9; 52.9)
		All	341	216	20.0 (13.0; 30.0)	330	227	28.8 (19.7; 40.7)	All	206	351	40.2 (27.2; 53.7)
**Naïve CD8⁺ T cells (% of CD8⁺ T cells)**	CCR7⁺, CD45RA⁺ of CD8⁺ T cells	RVd	165	114	29.0 (16.0; 44.0)	163	116	20.3 (11.4; 31.4)	RVd/R	37	102	7.7 (5.2; 11.3)
									RVd/Elo-R	56	84	10.1 (5.3; 18.4)
		Elo-RVd	176	102	26.4 (14.0; 42.2)	167	111	25.0 (12.5; 41.0)	Elo-RVd/R	56	81	7.5 (3.6; 12.6)
									Elo-RVd/Elo-R	57	84	12.2 (5.4; 20.0)
		All	341	216	27.2 (14.0; 43.0)	330	227	21.5 (11.9; 37.7)	All	206	351	9.1 (5.0; 16.0)
**Effector CD8⁺ T cells (% of CD8⁺ T cells)**	CCR7⁻, CD45RA⁺ of CD8⁺ T cells	RVd	165	114	26.0 (16.0; 40.0)	163	116	19.1 (11.0; 36.6)	RVd/R	37	102	31.2 (18.0; 42.0)
									RVd/Elo-R	56	84	16.6 (7.3; 37.0)
			176	102	26.3 (14.5; 43.2)	167	111	12.4 (7.5; 24.8)	Elo-RVd/R	56	81	29.1 (17.9; 47.8)

		Elo-RVd							Elo-RVd/Elo-R	57	84	15.1 (8.4; 36.9)
		All	341	216	26.0 (15.0; 41.0)	330	227	16.7 (8.3; 29.2)	All	206	351	22.8 (10.2; 42.0)
**Regulatory CD8^{+} T cells (% of CD8^{+} T cells)**	CD28^{-}, CD57^{+} of CD8^{+} T cells	RVd	173	106	26.2 (16.0; 40.0)	151	128	21.8 (10.1; 36.6)	RVd/R	36	103	25.1 (13.7; 45.6)
									RVd/Elo-R	56	84	14.3 (4.6; 30.6)
		Elo-RVd	176	102	26.8 (14.0; 41.3)	155	123	13.2 (4.4; 26.9)	Elo-RVd/R	56	81	35.4 (16.9; 49.4)
									Elo-RVd/Elo-R	56	85	9.1 (5.6; 33.8)
		All	349	208	26.3 (15.1; 40.8)	306	251	17.1 (7.7; 31.8)	All	204	353	22.0 (7.2; 43.0)

NA: not available; q1: first quantile; q3: third quantile.

Supplementary Table 3: Linear regression model on factors that influence effector $CD8^+$ T cell levels at T2

Linear regression comprising effector $CD8^+$ T cells at T1 (% of $CD8^+$ T cells), age, sex, and treatment arm as independent variables influencing the effector $CD8^+$ T cell levels at T2 (% of $CD8^+$ T cells).

Variable	Effect (change in % at T2)	95% CI	p-value
Effector $CD8^+$ T cells at T1 (per 10%)	5.34	4.47-6.21	**< 0.001**
Age (per 10 years)	-0.67	-2.67-1.34	0.51
Sex (female vs. male)	-0.375	-3.37-2.62	0.81
Treatment arm (Elo-RVd vs. RVd)	-9.027	-11.983- -6.07	**< 0.001**

Supplementary Table 4: Linear regression model on factors that influence regulatory $CD8^+$ T cell levels at T2

Linear regression comprising regulatory $CD8^+$ T cells at T1 (% of $CD8^+$ T cells), age, sex, and induction treatment as independent variables influencing the regulatory $CD8^+$ T cell levels at T2 (% of $CD8^+$ T cells).

Variable	Estimate (change in % at T2)	95% CI	p-value
Regulatory $CD8^+$ T cells at T1 (per 10%)	5.07	4.03-6.10	**< 0.001**
Age (per 10 years)	1.82	-0.38-4.02	0.11
Sex (female vs. male)	-3.44	-6.71- -0.17	**0.039**
Treatment arm (Elo-RVd vs. RVd)	-7.59	-10.80- -4.38	**< 0.001**

Supplementary Figure 1: Coordinate plots showing the percentages of T cell subpopulations and NK cells of each patient from inclusion (T1) to after induction therapy (T2) and from after induction therapy to consolidation and maintenance therapy (T3):

Coordinate plots of **A)** effector $CD8^+$ T cells, **B)** regulatory $CD8^+$ T cells, **C)** lymphocytes, **D)** NK cells, **E)** T cells, **F)** $CD4^+$ T cells, **G)** naïve $CD4^+$ T cells, **H)** central memory $CD4^+$ T cells, **I)** effector memory $CD4^+$ T cells, **J)** effector $CD4^+$ T cells, **K)** regulatory $CD4^+$ T cells, **L)** $CD8^+$ T cells, **M)** naïve $CD8^+$ T cells, **N)** central memory $CD8^+$ T cells, **O)** effector memory $CD8^+$ T cell frequency within study arm RVd/R compared to RVd/Elo-R and study arm Elo-RVd/R compared to Elo-RVd/Elo-R at T1, T2 and arm RVd/R, RVd/Elo-R, Elo-RVd/R and Elo-RVd/Elo-R at T3.

Differences between groups were evaluated using paired Student's t-test; $*$ p<0.05, $**$ p<0.01, $***$ p<0.001.

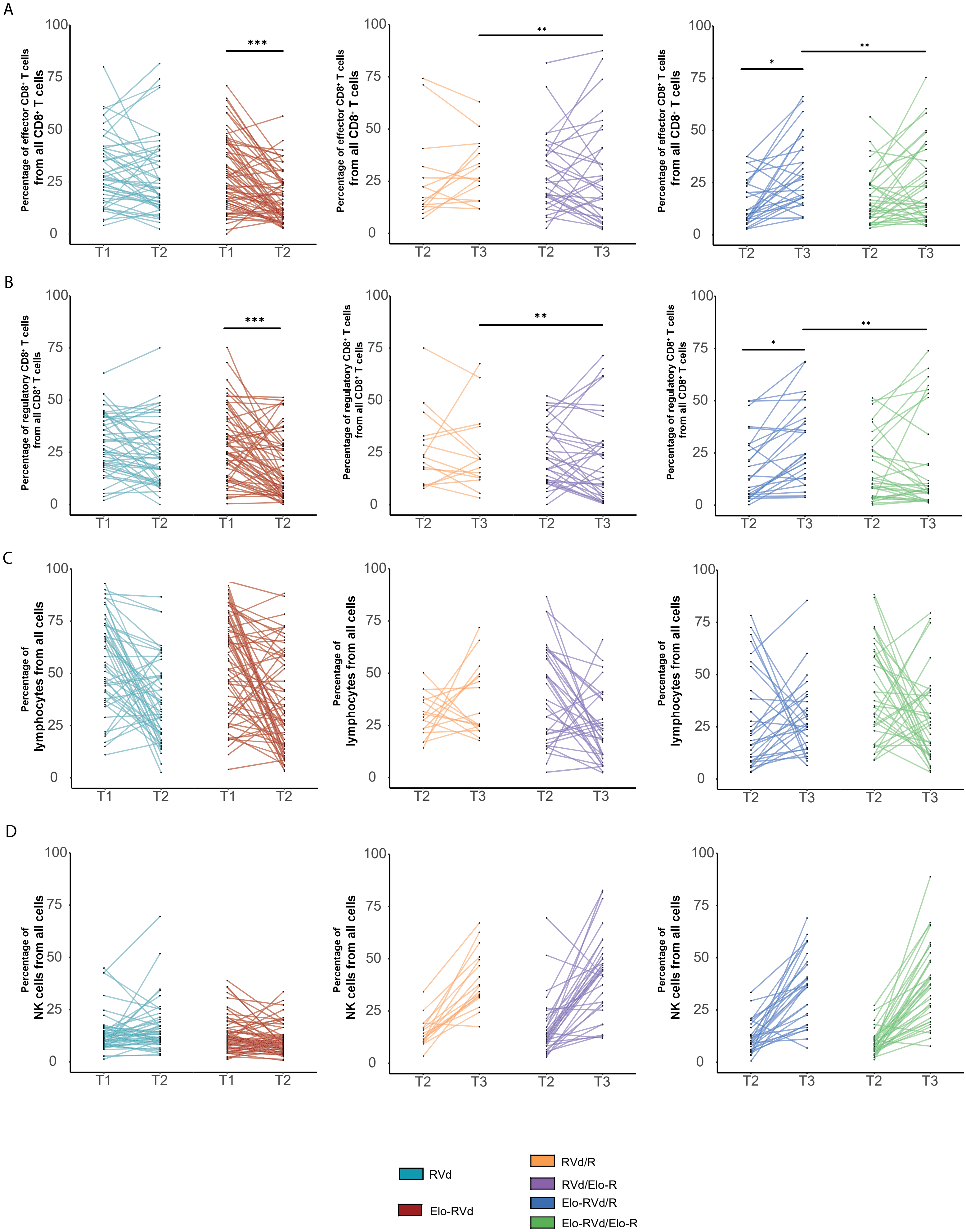
A
B
C
D
Percentage of effector CD8⁺ T cells from all CD8⁺ T cells
Percentage of regulatory CD8⁺ T cells from all CD8⁺ T cells
Percentage of lymphocytes from all cells
Percentage of NK cells from all cells
0
25
50
75
100
T1
T2
T3
***
**
*
RVd
Elo-RVd
RVd/R
RVd/Elo-R
Elo-RVd/R
Elo-RVd/Elo-R

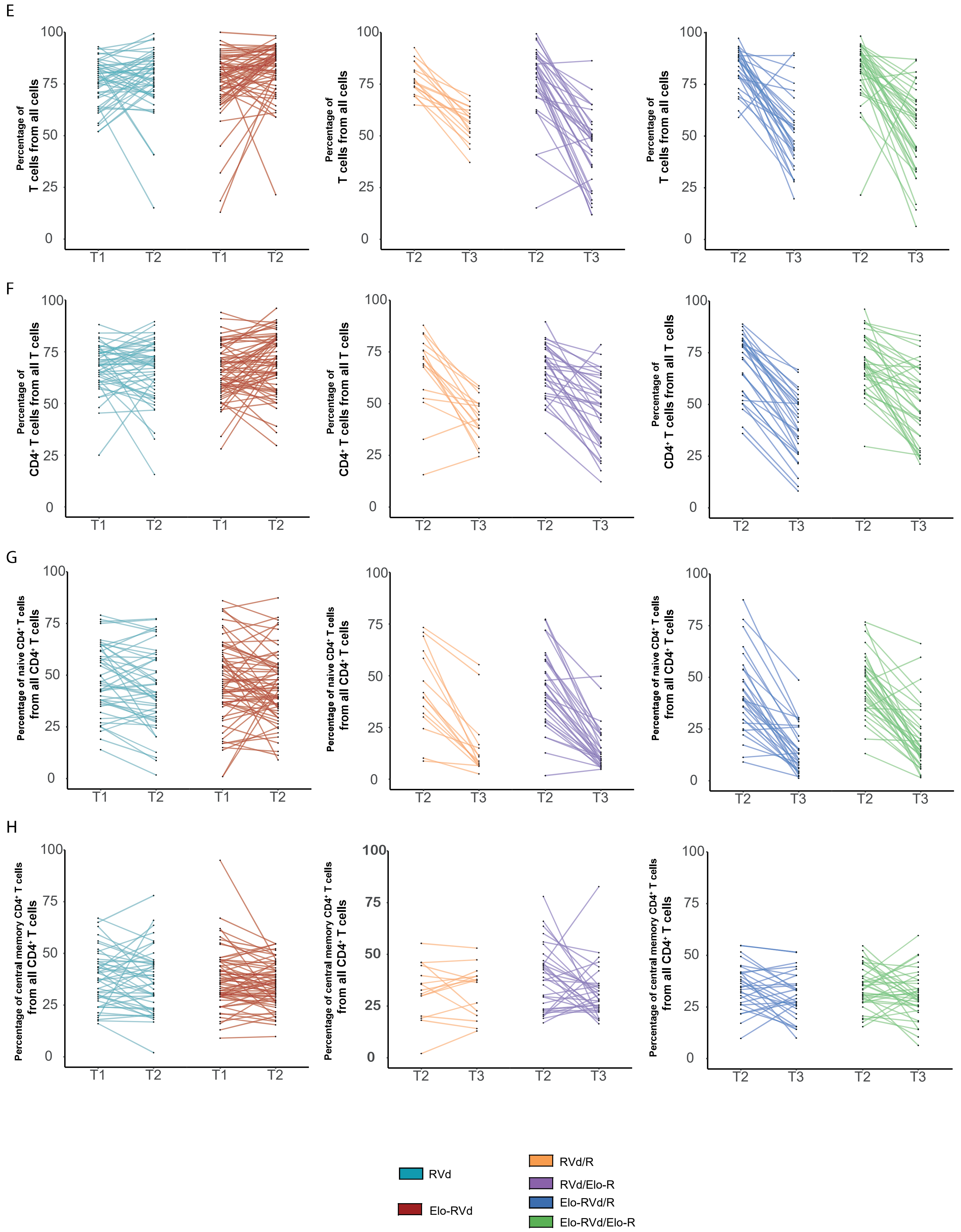
E
F
G
H
Percentage of T cells from all cells
Percentage of CD4⁺ T cells from all T cells
Percentage of naive CD4⁺ T cells from all CD4⁺ T cells
Percentage of central memory CD4⁺ T cells from all CD4⁺ T cells
0
25
50
75
100
T1
T2
T3
RVd
Elo-RVd
RVd/R
RVd/Elo-R
Elo-RVd/R
Elo-RVd/Elo-R

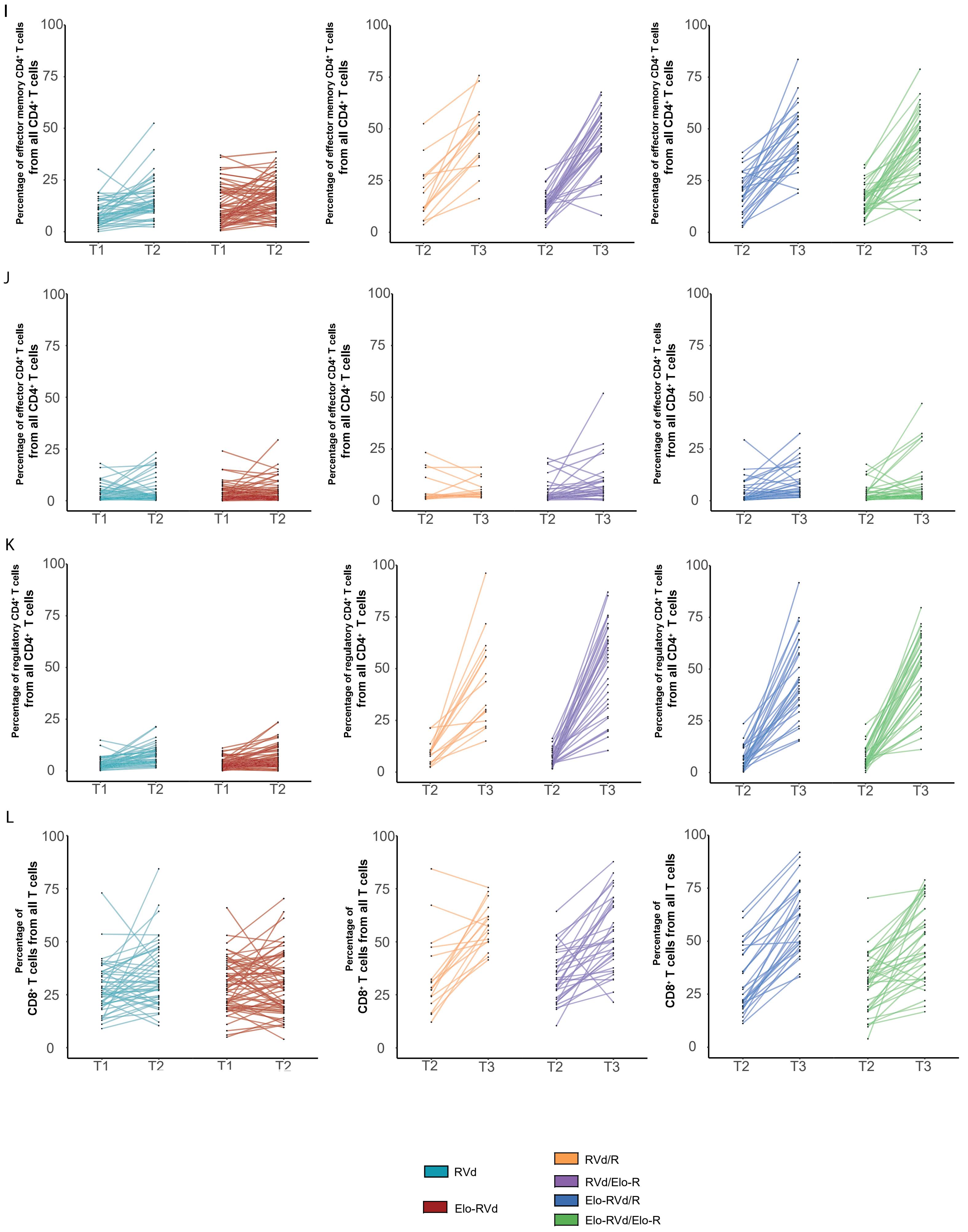
I
Percentage of effector memory CD4⁺ T cells from all CD4⁺ T cells
100
75
50
25
0
T1
T2
T3
J
Percentage of effector CD4⁺ T cells from all CD4⁺ T cells
K
Percentage of regulatory CD4⁺ T cells from all CD4⁺ T cells
L
Percentage of CD8⁺ T cells from all T cells
RVd
Elo-RVd
RVd/R
RVd/Elo-R
Elo-RVd/R
Elo-RVd/Elo-R

M

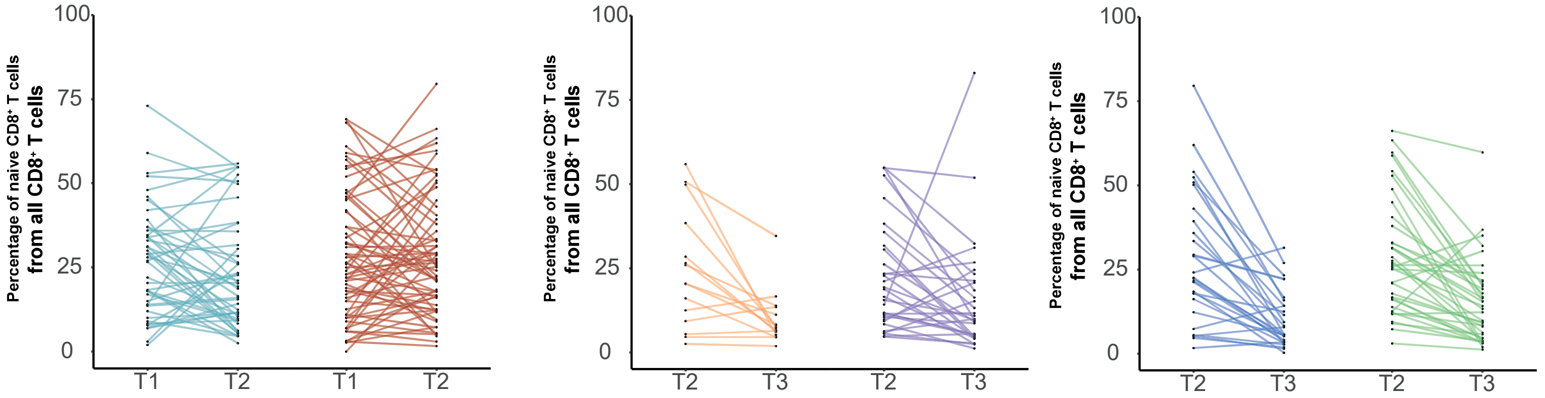
Percentage of naive CD8⁺ T cells from all CD8⁺ T cells
100
75
50
25
0
T1
T2
T1
T2
T3

N

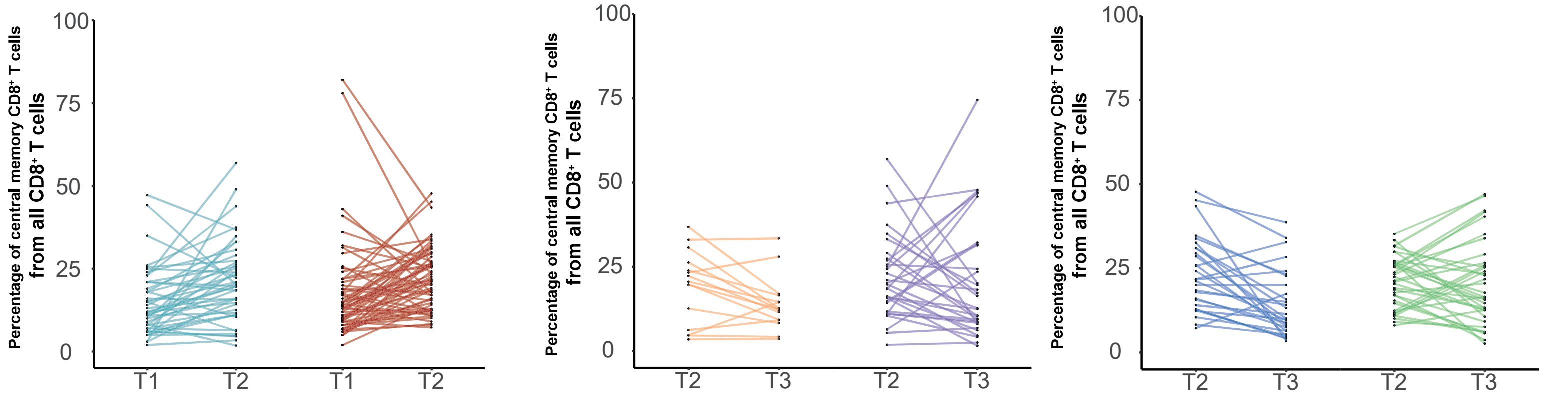
Percentage of central memory CD8⁺ T cells from all CD8⁺ T cells
100
75
50
25
0
T1
T2
T1
T2
T3

O

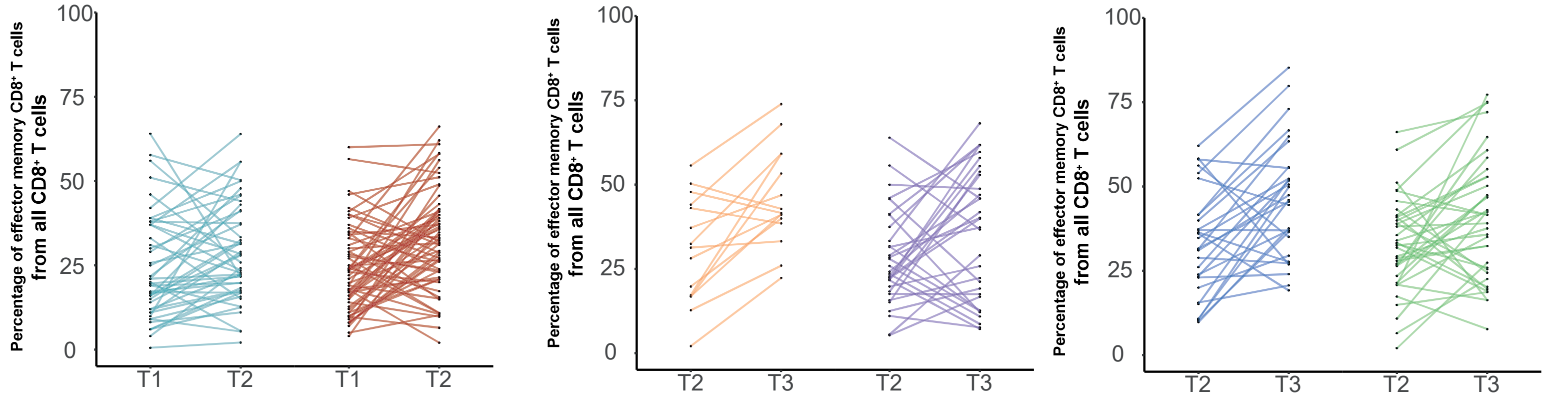
Percentage of effector memory CD8⁺ T cells from all CD8⁺ T cells
100
75
50
25
0
T1
T2
T1
T2
T3

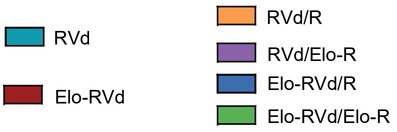
RVd
Elo-RVd
RVd/R
RVd/Elo-R
Elo-RVd/R
Elo-RVd/Elo-R

Supplementary Figure 2: Flow cytometry analysis of SLAMF7 expression in effector $CD8^+$ and regulatory $CD8^+$ T cells of MM patients:

(A) Bar plot and **(B)** a representative histogram showing MFI of SLAMF7 expression in effector $CD8^+$ T cells compared to non-effector $CD8^+$ T cells in BM samples from MM patients at T1 (n = 5). **(C)** Bar plot and **(D)** a representative histogram showing MFI of SLAMF7 expression in regulatory $CD8^+$ T cells compared to non-regulatory $CD8^+$ T cell compartment in BM samples from MM patients at T1 (n = 5). Bar plots showing MFI of SLAMF7 expression in **(E)** effector $CD8^+$ T cells (T1: n = 5, T2 n = 5, T3: n = 5) and **(F)** regulatory $CD8^+$ T cells (T1: n = 5, T: n = 10, T3: n = 5) in PB samples of MM patients at time points T1, T2 and T3.

*Differences between groups were evaluated using paired Student's t-test; * p<0.05, ** p<0.01, *** p<0.001.*

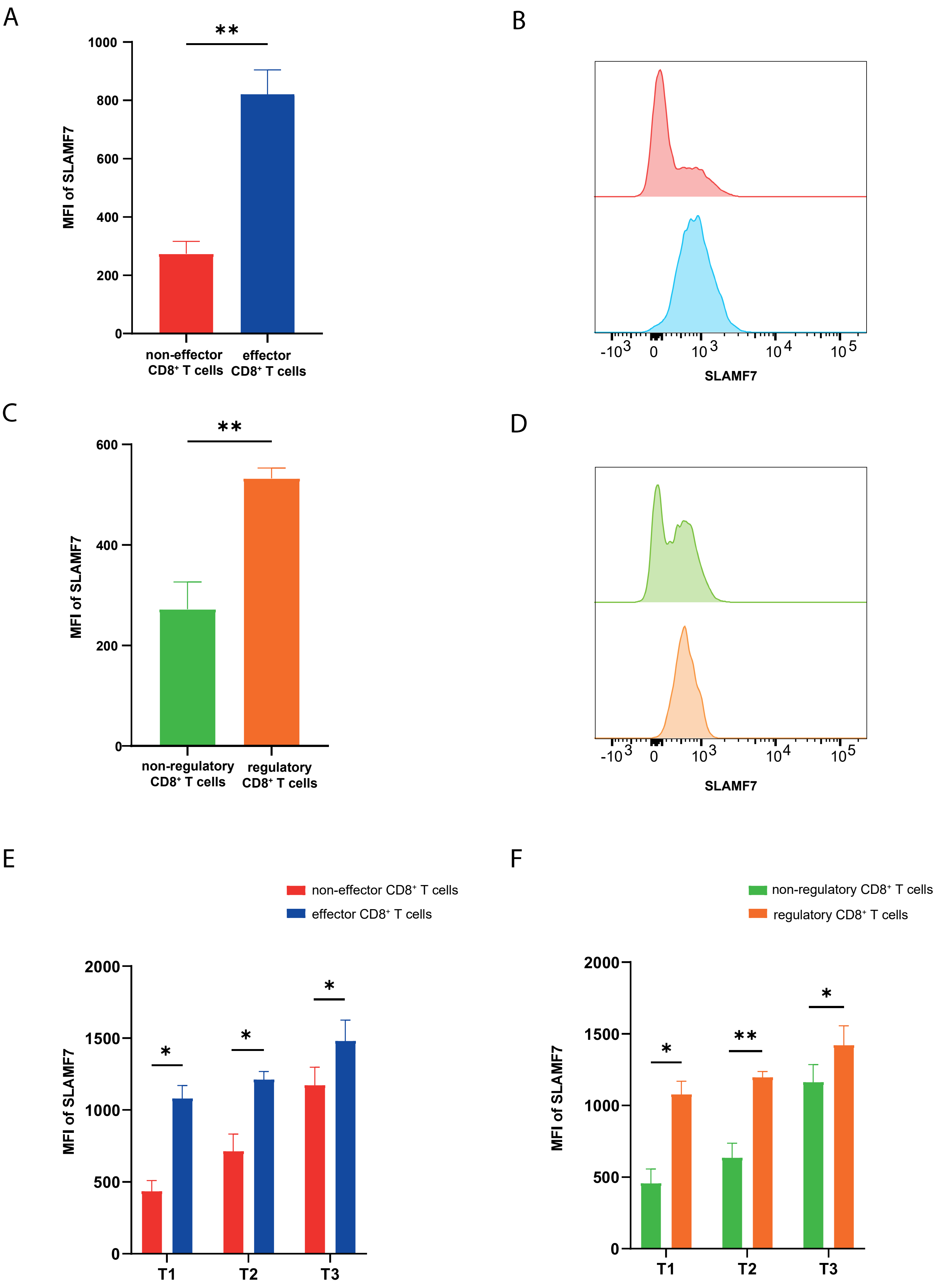
A
**
MFI of SLAMF7
1000
800
600
400
200
0
non-effector CD8⁺ T cells
effector CD8⁺ T cells
B
-10 3
0
10 3
10 4
10 5
SLAMF7
C
**
MFI of SLAMF7
600
400
200
0
non-regulatory CD8⁺ T cells
regulatory CD8⁺ T cells
D
-10 3
0
10 3
10 4
10 5
SLAMF7
E
non-effector CD8⁺ T cells
effector CD8⁺ T cells
MFI of SLAMF7
2000
1500
1000
500
0
*
*
*
T1
T2
T3
F
non-regulatory CD8⁺ T cells
regulatory CD8⁺ T cells
MFI of SLAMF7
2000
1500
1000
500
0
*
**
*
T1
T2
T3

Supplementary Figure 3: Dot plots and pie charts showing the percentages of effector $CD8^+$ T cells, regulatory $CD8^+$ T cells and other $CD8^+$ T cell subsets of MM patients from inclusion (T1), after induction therapy (T2) and during consolidation and maintenance therapy (T3) by treatment arms:

Dot plots of the percentages of **A)** effector $CD8^+$ T cells and **B)** regulatory $CD8^+$ T cells ($CD8^+$ $CD28^-$) from lymphocytes at T1 and T2 in study arm RVd and Elo-RVd. **C)** & **D)** The percentage of effector $CD8^+$ T cells from lymphocytes at T2 and T3 in study arms RVd/R, RVd/Elo-R, Elo-RVd/R and Elo-RVd/Elo-R. **E)** & **F)** The percentage of regulatory $CD8^+$ T ($CD8^+$ $CD28^-$) cells from lymphocytes at T2 and T3 in study arms RVd/R, RVd/Elo-R, Elo-RVd/R and Elo-RVd/Elo-R. Pie charts of the median percentage of effector, effector memory, naïve and central memory $CD8^+$ T cell subset from all $CD8^+$ T cells **G)** at T1 and T2 in study arm RVd, **H)** at T1 and T2 in study arm Elo-RVd, **I)** at T2 and T3 in study arm RVd/R and RVd/Elo-R and **J)** at T2 and T3 in study arm Elo-RVd/R and Elo-RVd/Elo-R.

*Differences between groups were evaluated using paired Student's t-test; * $p<0.05$, ** $p<0.01$, *** $p<0.001$.*

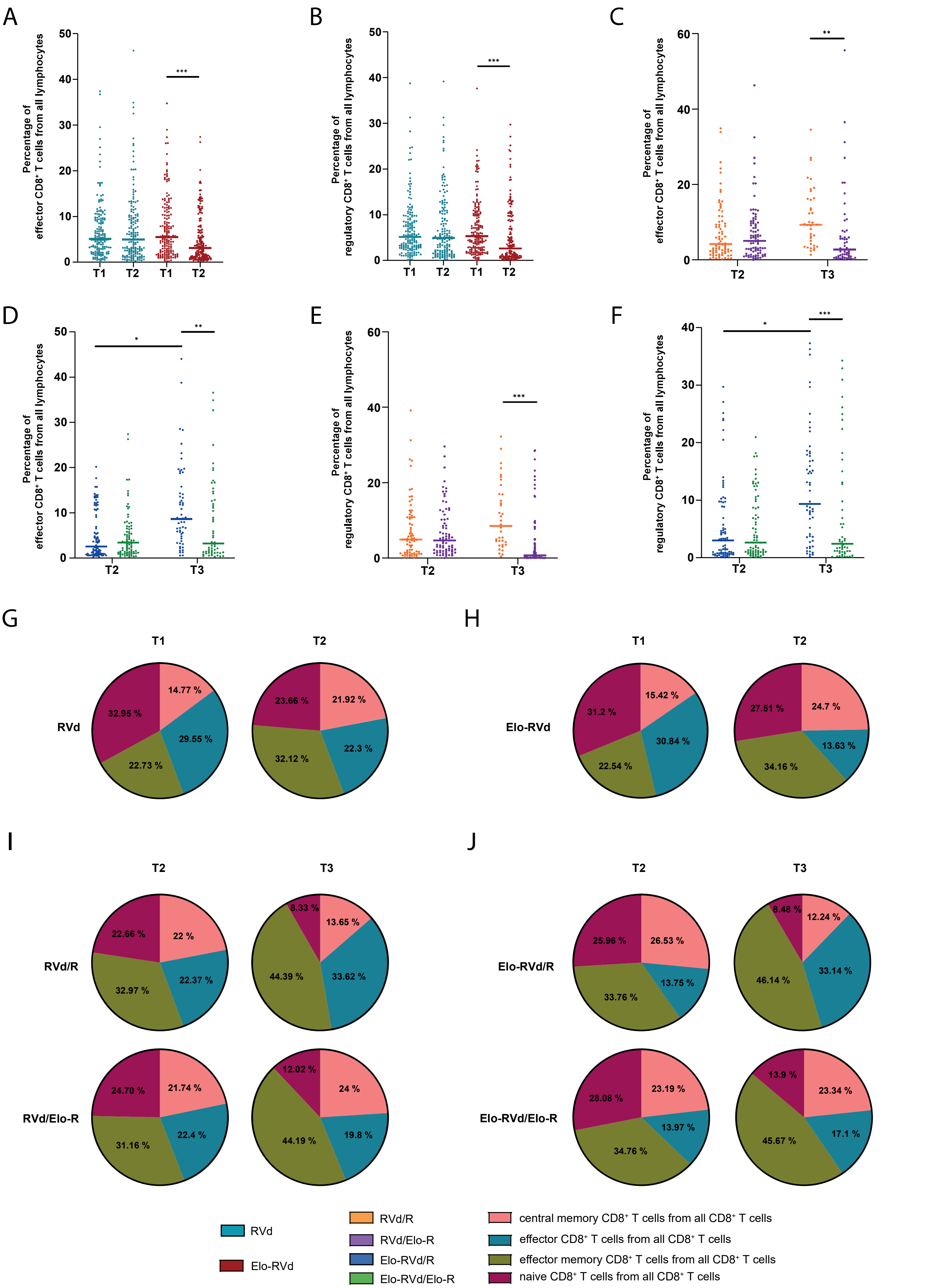
A
B
C
D
E
F
G
H
I
J
Percentage of effector CD8⁺ T cells from all lymphocytes
Percentage of regulatory CD8⁺ T cells from all lymphocytes
T1
T2
T3
RVd
Elo-RVd
RVd/R
RVd/Elo-R
Elo-RVd/R
Elo-RVd/Elo-R
14.77 %
32.95 %
29.55 %
22.73 %
23.66 %
21.92 %
32.12 %
22.3 %
15.42 %
31.2 %
30.84 %
22.54 %
27.51 %
24.7 %
13.63 %
34.16 %
22.66 %
22 %
32.97 %
22.37 %
8.33 %
13.65 %
44.39 %
33.62 %
25.96 %
26.53 %
13.75 %
33.76 %
8.48 %
12.24 %
46.14 %
33.14 %
24.70 %
21.74 %
31.16 %
22.4 %
12.02 %
24 %
44.19 %
19.8 %
28.08 %
23.19 %
13.97 %
34.76 %
13.9 %
23.34 %
45.67 %
17.1 %
RVd
Elo-RVd
RVd/R
RVd/Elo-R
Elo-RVd/R
Elo-RVd/Elo-R
central memory CD8⁺ T cells from all CD8⁺ T cells
effector CD8⁺ T cells from all CD8⁺ T cells
effector memory CD8⁺ T cells from all CD8⁺ T cells
naive CD8⁺ T cells from all CD8⁺ T cells

Supplementary Figure 4: Kaplan–Meier curves showing progression-free survival of patients, evaluating the abundance of regulatory CD8$^+$ (CD8$^+$ CD28$^-$) T cells after induction therapy (T2), improving the prediction of response to elotuzumab-based maintenance therapy:

Kaplan–Meier curves of **A)** high regulatory CD8$^+$ T cells at T2: arm [RVd/Elo-R versus RVd/R], arm [Elo-RVd/R versus RVd/R], and arm [Elo-RVd/Elo-R versus RVd/R] and **B)** Low regulatory CD8$^+$ T cells at T2: arm [RVd/Elo-R versus RVd/R]. **C)** Arm RVd/R: regulatory CD8$^+$ T cells at T2 [high versus low]. **D)** Arm Elo-RVd/R: regulatory CD8$^+$ T cells at T2 [high versus low]. **E)** Arm RVd/Elo-R: regulatory CD8$^+$ T cells at T2 [high versus low]. **F)** Arm Elo-RVd/Elo-R: regulatory CD8$^+$ T cells at T2 [high versus low].

A

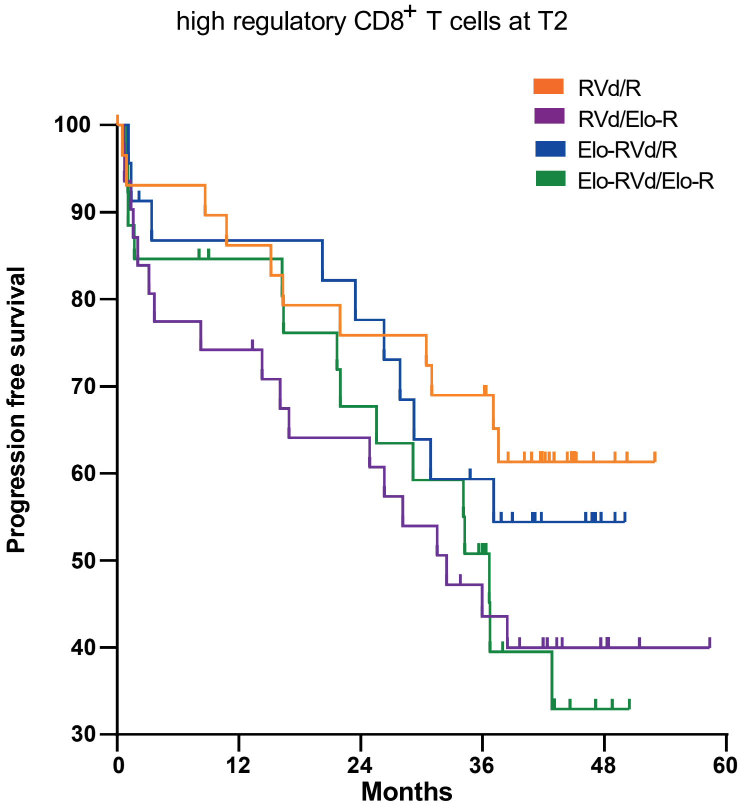

RVd/R	36	30	27	23	4	0
RVd/Elo-R	38	29	25	16	4	0
Elo-RVd/R	26	22	20	14	2	0
Elo-RVd/Elo-R	27	21	17	12	2	0

B

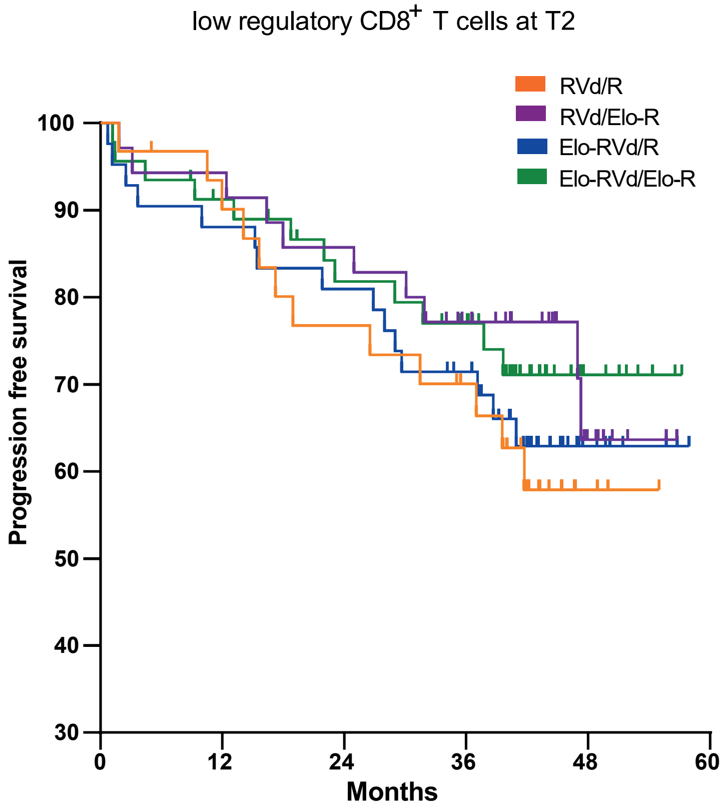

RVd/R	35	30	26	22	3	0
RVd/Elo-R	41	39	36	28	8	0
Elo-RVd/R	51	45	40	33	9	0
Elo-RVd/Elo-R	49	43	37	33	9	0

low regulatory CD8^{+} T cells   high regulatory CD8^{+} T cells

C

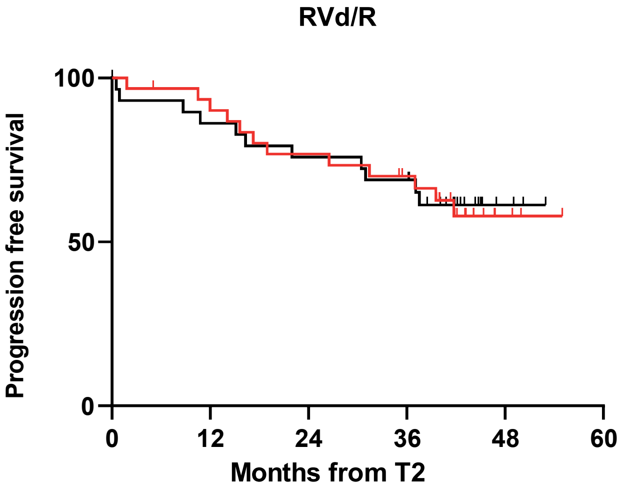

Low	35	30	26	22	3	0
High	36	30	27	23	4	0

D

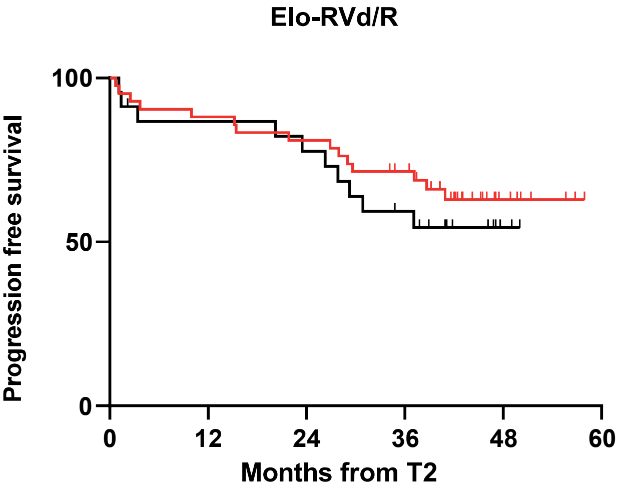

Low	51	45	40	33	9	0
High	26	22	20	14	2	0

E

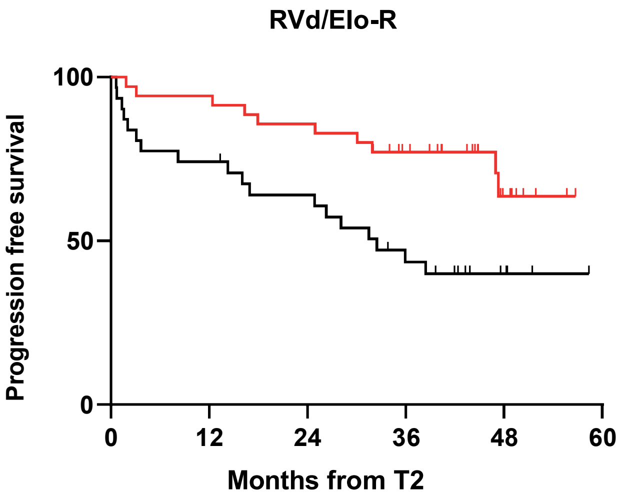

Low	41	39	36	28	8	0
High	38	29	25	16	4	0

F

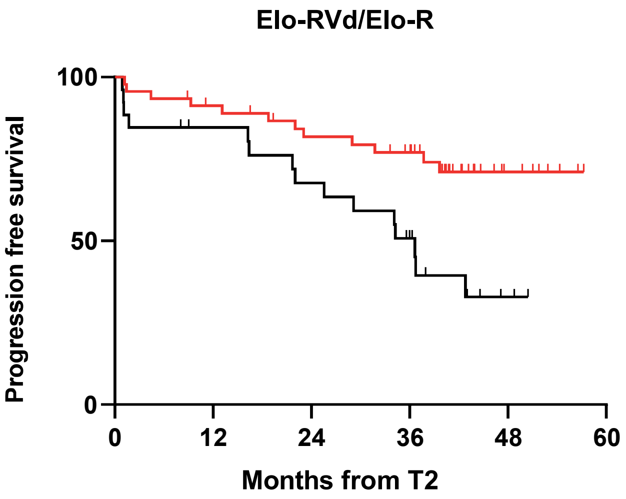

Low	49	43	37	33	9	0
High	27	21	17	12	2	0

Supplementary Figure 5: Gating strategy for monitoring T cell subpopulations and NK cell percentages:

T cells were identified after removal of **A)** debris and **B)** doublets as **C)** lymphocytes and among lymphocytes as **D)** $CD3^+$ cells and **E)** NK cells as $CD3^-$ and $CD56^+$ cells. **F)** $CD4^+$ T-cell subpopulations were identified among $CD4^+$ T cells according to the expression of CCR7 and CD45RA as follows: **G)** central memory ($CCR7^+$, $CD45RA^-$), effector memory ($CCR7^-$, $CD45RA^-$), naïve ($CCR7^+$, $CD45RA^+$), and effector ($CCR7^-$, $CD45RA^+$) $CD4^+$ T cells. **H)** Regulatory $CD4^+$ T cells were defined as $CD25^+$ cells. **I)** $CD8^+$ T-cell subpopulations were identified according to the expression of CCR7 and CD45RA as follows: **J)** central memory ($CCR7^+$, $CD45RA^-$), effector memory ($CCR7^-$, CD45RA-), naïve ($CCR7^+$, $CD45RA^+$), and effector ($CCR7^-$, $CD45RA^+$) $CD8^+$ T cells. **K)** Regulatory $CD8^+$ T cells were defined as $CD28^-$ and $CD57^+$ cells.

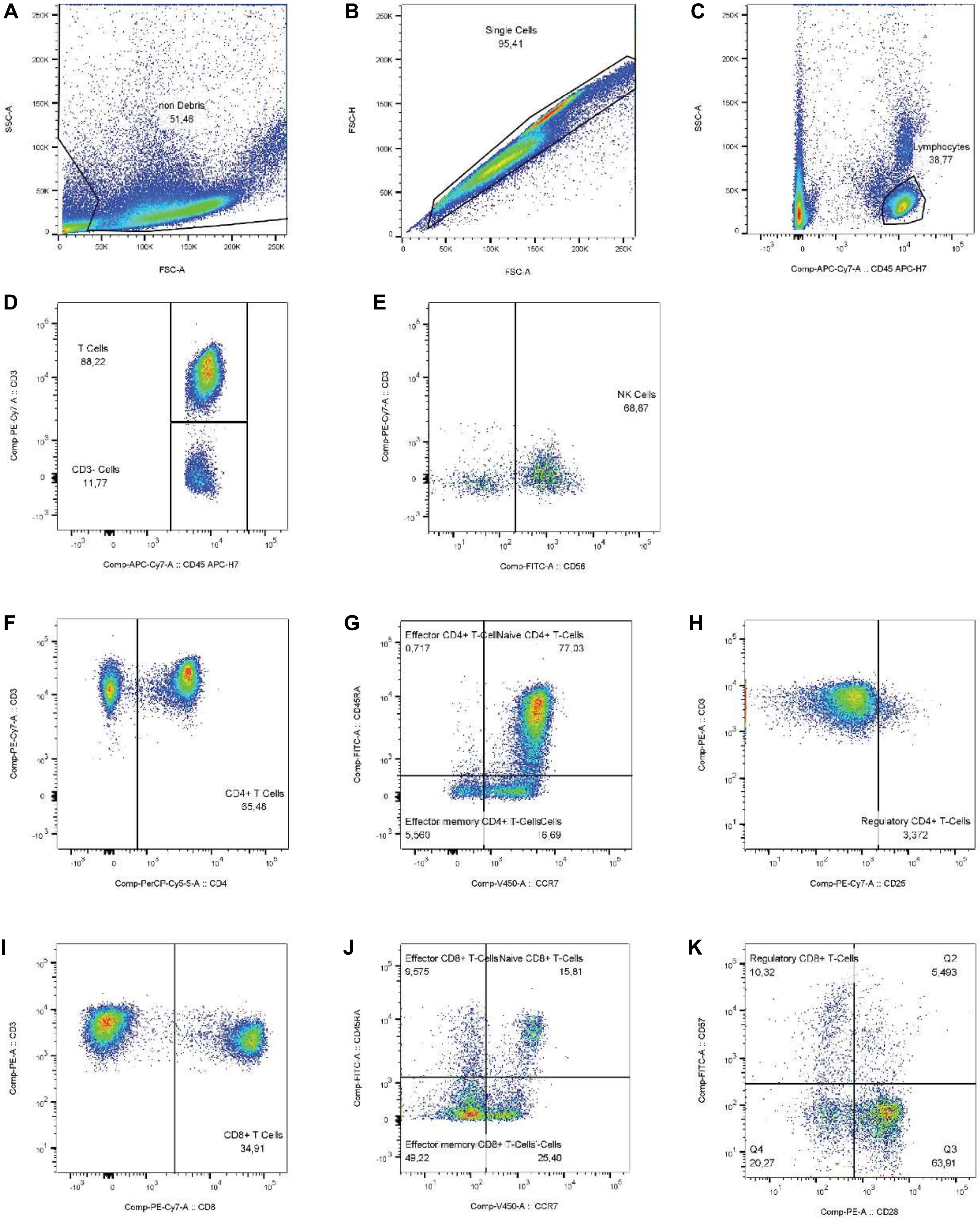
A
non Debris
51,46
SSC-A
FSC-A
B
Single Cells
95,41
FSC-H
FSC-A
C
Lymphocytes
38,77
SSC-A
Comp-APC-Cy7-A :: CD45 APC-H7
D
T Cells
88,22
CD3- Cells
11,77
Comp-PE-Cy7-A :: CD3
Comp-APC-Cy7-A :: CD45 APC-H7
E
NK Cells
68,87
Comp-PE-Cy7-A :: CD3
Comp-FITC-A :: CD56
F
CD4+ T Cells
65,48
Comp-PE-Cy7-A :: CD3
Comp-PerCP-Cy5-5-A :: CD4
G
Effector CD4+ T-CellNaive CD4+ T-Cells
0,717
77,03
Effector memory CD4+ T-CellsCells
5,560
16,69
Comp-FITC-A :: CD45RA
Comp-V450-A :: CCR7
H
Regulatory CD4+ T-Cells
3,372
Comp-PE-A :: CD3
Comp-PE-Cy7-A :: CD25
I
CD8+ T Cells
34,91
Comp-PE-A :: CD3
Comp-PE-Cy7-A :: CD8
J
Effector CD8+ T-CellsNaive CD8+ T-Cells
9,575
15,81
Effector memory CD8+ T-Cells'-Cells
49,22
25,40
Comp-FITC-A :: CD45RA
Comp-V450-A :: CCR7
K
Regulatory CD8+ T-Cells
10,32
Q2
5,493
Q4
20,27
Q3
63,91
Comp-FITC-A :: CD57
Comp-PE-A :: CD28

References

[1] H. Salwender, U. Bertsch, K. Weisel, J. Dürig, C. Kunz, A. Benner et al., “Rationale and design of the German-speaking myeloma multicenter group (GMMG) trial HD6: a randomized phase III trial on the effect of elotuzumab in VRD induction/consolidation and lenalidomide maintenance in patients with newly diagnosed myeloma,” *BMC cancer*, vol. 19, no. 1, p. 504, 2019.

[2] H. Goldschmidt, E. K. Mai, U. Bertsch, B. Besemer, M. Haenel, K. Miah et al., “Elotuzumab in Combination with Lenalidomide, Bortezomib, Dexamethasone and Autologous Transplantation for Newly-Diagnosed Multiple Myeloma: Results from the Randomized Phase III GMMG-HD6 Trial,” *Blood*, vol. 138, Supplement 1, p. 486, 2021.

[3] H. Goldschmidt, E. K. Mai, J. Dürig, C. Scheid, K. C. Weisel, C. Kunz et al., “Response-adapted lenalidomide maintenance in newly diagnosed myeloma: results from the phase III GMMG-MM5 trial,” *Leukemia*, vol. 34, no. 7, pp. 1853–1865, 2020.

[4] S. Wuilleme, N. Robillard, L. Lodé, F. Magrangeas, H. Beris, J-L. Harousseau et al., “Ploidy, as detected by fluorescence in situ hybridization, defines different subgroups in multiple myeloma,” *Leukemia*, vol. 19, no. 2, pp. 275–278, 2005.

[5] P. Sonneveld, H. Avet-Loiseau, S. Lonial, S. Usmani, D. Siegel, K. C. Anderson et al., “Treatment of multiple myeloma with high-risk cytogenetics: a consensus of the International Myeloma Working Group,” *Blood*, vol. 127, no. 24, pp. 2955–2962, 2016.

[6] B. Lausen and M. Schumacher, “Maximally Selected Rank Statistics,” *Biometrics*, vol. 48, no. 1, p. 73, 1992.

[7] E. L. Kaplan and P. Meier, “Nonparametric Estimation from Incomplete Observations,” *Journal of the American Statistical Association*, vol. 53, no. 282, p. 457, 1958.

[8] P. R. Greipp, J. San Miguel, B. G. M. Durie, J. J. Crowley, B. Barlogie, J. Bladé et al., “International staging system for multiple myeloma,” *Journal of Clinical Oncology*, vol. 23, no. 15, pp. 3412–3420, 2005.

[9] A. Palumbo, H. Avet-Loiseau, S. Oliva, H. M. Lokhorst, H. Goldschmidt, L. Rosinol et al., “Revised International Staging System for Multiple Myeloma: A Report From International Myeloma Working Group,” *Journal of clinical oncology : official journal of the American Society of Clinical Oncology*, vol. 33, no. 26, pp. 2863–2869, 2015.
